# Optimized infrared photoactivatable ribonucleoside-enhanced crosslinking and immunoprecipitation (IR-PAR-CLIP) protocol identifies novel IGF2BP3-interacting RNAs in colon cancer cells

**DOI:** 10.1261/rna.079714.123

**Published:** 2023-11

**Authors:** Aleksandra S. Anisimova, G. Elif Karagöz

**Affiliations:** 1 Max Perutz Labs, Vienna BioCenter Campus (VBC), 1030 Vienna, Austria; 2 Medical University of Vienna, Center for Medical Biochemistry, 1030 Vienna, Austria; 3 Vienna BioCenter PhD Program, a Doctoral School of the University of Vienna and the Medical University of Vienna, 1030 Vienna, Austria

**Keywords:** IGF2BP3, PAR-CLIP, RNA stability, posttranscriptional regulation

## Abstract

The conserved family of RNA-binding proteins (RBPs), IGF2BPs, plays an essential role in posttranscriptional regulation controlling mRNA stability, localization, and translation. Mammalian cells express three isoforms of IGF2BPs: IGF2BP1-3. IGF2BP3 is highly overexpressed in cancer cells, and its expression correlates with a poor prognosis in various tumors. Therefore, revealing its target RNAs with high specificity in healthy tissues and in cancer cells is of crucial importance. Photoactivatable-ribonucleoside-enhanced crosslinking and immunoprecipitation (PAR-CLIP) identifies the binding sites of RBPs on their target RNAs at nucleotide resolution in a transcriptome-wide manner. Here, we optimized the PAR-CLIP protocol to study RNA targets of endogenous IGF2BP3 in a human colorectal carcinoma cell line. To this end, we first established an immunoprecipitation protocol to obtain highly pure endogenous IGF2BP3–RNA complexes. Second, we modified the protocol to use highly sensitive infrared (IR) fluorescent dyes instead of radioactive probes to visualize IGF2BP3-crosslinked RNAs. We named the modified method “IR-PAR-CLIP.” Third, we compared RNase cleavage conditions and found that sequence preferences of the RNases impact the number of the identified IGF2BP3 targets and introduce a systematic bias in the identified RNA motifs. Fourth, we adapted the single adapter circular ligation approach to increase the efficiency in library preparation. The optimized IR-PAR-CLIP protocol revealed novel RNA targets of IGF2BP3 in a human colorectal carcinoma cell line. We anticipate that our IR-PAR-CLIP approach provides a framework for studies of other RBPs.

## INTRODUCTION

RNA-binding proteins (RBPs) play a crucial role in the posttranscriptional regulation of gene expression. They regulate fundamental steps in the RNA life cycle including RNA splicing, stabilization, subcellular localization, translation, and degradation (Nielsen et al. 1999; Ladd et al. 2001; Fallini et al. 2011; Mizutani et al. 2016). The insulin-like growth factor 2 mRNA-binding proteins (IGF2BPs/IMPs) are a family of RBPs conserved from insects to mammals (Bell et al. 2013). Originally IGF2BPs were identified as posttranscriptional regulators of mRNA encoding for growth factor IGF2 (Nielsen et al. 1999; Zhang et al. 1999). Mammals have three IGF2BP paralogs (IGF2BP1-3), which are oncofetal proteins expressed during early development and in various cancers. Their crucial role in early development was shown in Xenopus (Yaniv et al. 2003), mice (Hansen et al. 2004), and zebrafish (Ren et al. 2020; Vong et al. 2021). Whereas the expression of IGF2BP1/3 decreases in most adult tissues, IGF2BP2 retains its expression and was shown to regulate lipid and glucose metabolism in adults (Hansen et al. 2004; Hammer et al. 2005; Bell et al. 2013; Laggai et al. 2014; Dai et al. 2015; Regué et al. 2019; Lu et al. 2021).

Due to its overexpression in aggressive tumors, IGF2BP3 is currently heavily studied. IGF2BP3 was initially identified as a highly overexpressed gene in pancreatic cancer (Müeller-Pillasch et al. 1997). In addition to pancreatic cancers, IGF2BP3 is highly expressed in various cancers including lung, liver, breast, skin, and colon (Samanta et al. 2013; Zhao et al. 2017; Xu et al. 2019; Hanniford et al. 2020; Huang et al. 2020). Its overexpression is strongly correlated with tumor aggressiveness and poor patient prognosis (Ross et al. 2001; Dimitriadis et al. 2007). IGF2BP3 shuttles between the nucleus and cytosol (Rivera Vargas et al. 2014), yet it is mainly found in the cytosol. It regulates the stability of oncogenic mRNAs *MYC* and *HMGA2* (Jønson et al. 2014; Huang et al. 2018) and controls the protein levels of cyclins D1, D3, and G1 (Rivera Vargas et al. 2014). This regulation was suggested to promote cell proliferation and tumor growth. Moreover, since IGF2BP3 binds to a large number of mRNAs in cells, it is likely that it controls the stability of mRNAs participating in various pathways involved in cellular homeostasis, thereby additionally contributes to tumorigenesis. Therefore, identifying RNAs interacting with IGF2BPs in a transcriptome-wide manner is crucial. IGF2BP paralogs share a high sequence identity in the amino acid level (∼60% among three paralogs). The sequence identity reaches 73% between IGF2BP1 and IGF2BP3 paralogs. Currently, the functional differences in IGF2BP paralogs remain largely uncovered.

Genome-wide crosslinking and immunoprecipitation (CLIP) methods have been instrumental in identifying the RNA targets of various RBPs. CLIP methods rely on in vivo photo-crosslinking of proteins to RNAs in cells followed by the immunoprecipitation of RBPs of interest to identify RNAs directly interacting with those RBPs (Lee and Ule 2018; Hafner et al. 2021). Over the years, several variations of CLIP methods have been introduced to increase the stringency, efficiency, and resolution of those approaches. The high-throughput sequencing of RNA isolated by crosslinking immunoprecipitation (HITS-CLIP) for the first time implemented the use of deep sequencing in the CLIP approaches allowing for genome-wide identification of RBP-binding sites in RNAs (Licatalosi et al. 2008). To increase the resolution in identifying RBP-binding sites in RNAs, alternative CLIP strategies were developed enabling precise mapping of the RBP-binding sites in their target RNAs at nucleotide resolution. The photoactivatable-ribonucleoside-enhanced crosslinking and immunoprecipitation (PAR-CLIP) relies on identifying the mutations introduced by the reverse transcriptase at the crosslink sites. In contrast, the individual-nucleotide resolution UV crosslinking and immunoprecipitation (iCLIP) leverages the termination of reverse transcription at the peptide–RNA crosslink sites (König et al. 2010).

PAR-CLIP methods revealed a large overlap of RNA targets of IGF2BP paralogs in human embryonic kidney (HEK) 293 cells (Hafner et al. 2010). In human pluripotent stem cells, a modified version of iCLIP, with improved library preparation, referred to as enhanced CLIP (eCLIP) (Van Nostrand et al. 2016), showed that while IGF2BP1 and IGF2BP2 were bound to a highly similar group of RNAs, IGF2BP3 displayed a binding preference that was distinct from the other paralogs (Conway et al. 2016). These results indicated that the paralogs play both redundant and distinct functions during early development and in different tissues. However, systematic characterization of RNA binding of the IGF2BP paralogs across cell types and tissues remains largely unexplored.

In recent years, significant improvements were made in the multistep CLIP methods, and several modifications were made to the protocols to overcome various challenges during the library preparation (Lee and Ule 2018; Hafner et al. 2021). Due to low input amounts, originally radioisotopes were used in these methods to visualize the RNA. Currently, the 3′ adapter conjugated to the fluorescent or infrared (IR) dye is being used to avoid radioactivity making these methods more accessible (Zarnegar et al. 2016; Kaczynski et al. 2019; Anastasakis et al. 2021). The low RNA input amounts represent a major challenge for the CLIP methods. To overcome this, increasing the efficiency of the library construction has been crucial. In addition to the low RNA input, the inefficient readthrough of the oligopeptide crosslink sites by the reverse transcriptase presents another challenge. The use of highly processive reverse transcriptases was shown to increase the efficiency of the library preparation and to produce libraries with higher complexity (Zarnegar et al. 2016; Van Nostrand et al. 2017). As the major goal of the CLIP methods is the identification of the RBP-binding sites with high precision, the interpretation of the results is highly influenced by the sequence bias introduced during small RNA library preparation. One of the sources of the sequence bias is the ligation of the adapters (Hafner et al. 2011). The single adapter strategy combined with circular ligation and the addition of the short random sequences to the 5′ ends of the adapter were shown to be effective in reducing sequence bias at this step in iCLIP (König et al. 2010), miRNA (Hafner et al. 2011; Barberán-Soler et al. 2018), and ribosome profiling (Lecanda et al. 2016) libraries. Another source of sequence bias in CLIP experiments is the selection of RNase treatment conditions to obtain short RNA sequences to precisely map the RBP-binding sites (Kishore et al. 2011). Importantly, different RNases were shown to produce very distinct read coverage profiles in ribosome profiling experiments, which rely on the mapping of short RNase-digested footprints similar to the CLIP methods (Gerashchenko and Gladyshev 2017). Therefore, the RNase selection and treatment conditions have to be carefully assessed and optimized.

To date, PAR-CLIP approaches have been instrumental in identifying RNAs interacting with several important RBPs in the cell (Hafner et al. 2010; Ascano et al. 2012; Gregersen et al. 2014). The PAR-CLIP relies on the incorporation of photoactivatable modified nucleoside analogs (4-thiouridine [4sU] or 6-thioguanine [6SG]) into cellular RNAs. Under UV light (365 nm) photoactivatable nucleoside analogs, 4sU being the most commonly used, covalently crosslink with the interacting proteins. The 4sU incorporation leads to 100- to 1000-fold increased crosslinking efficiency compared to the UV crosslinking at 254 nm. The protein–RNA complexes are then immunoprecipitated in combination with the two-step RNase treatment: in-lysate and on-beads RNase to shorten the RNA fragments, and the protein is digested with proteinase K to remove the polypeptide. The peptide remnants of the protein at the crosslink site result in the T to C transitions in the final sequencing library allowing identification of the RBP-binding sites with single-nucleotide resolution (Hafner et al. 2010). Here, we describe a modified PAR-CLIP protocol optimized for immunoprecipitation of the endogenous human IGF2BP3 in colon carcinoma cell lines. Our modified PAR-CLIP strategy uses the IR-labeled 3′ adapter and circular ligation to increase the accessibility of the method and to decrease bias in library preparation. Importantly, by comparing the IGF2BP3-target transcripts identified in samples treated with different RNases, we revealed that RNase selection is crucial for both the identification of certain targets and the prediction of the RBP-binding sites. We anticipate that our modified PAR-CLIP protocol can be utilized for characterizing IGF2BP3 targets as well as studying other RBPs in various tissues and cancer cells.

## RESULTS

### Overview of the infrared PAR-CLIP protocol

In this work, we present a modified version of the PAR-CLIP protocol (Hafner et al. 2010; Danan et al. 2016). We optimized three crucial aspects in the protocol: (i) the immunoprecipitation (IP) of the endogenous protein (on the example of IGF2BP3), (ii) RNase treatment conditions to reduce sequence bias, and (iii) increasing the safety and efficiency of the protocol.

**FIGURE 1.**
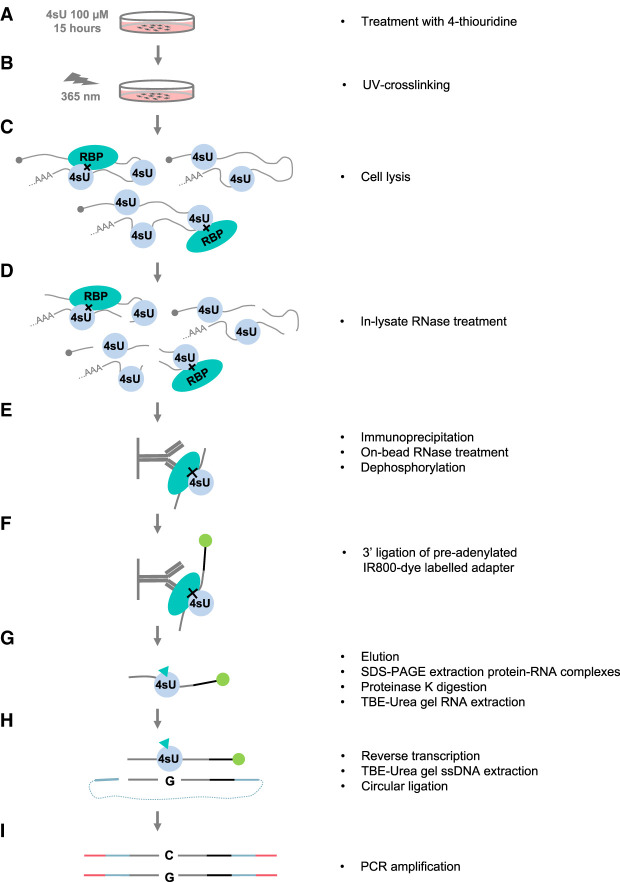
Schematic of the infrared PAR-CLIP. (*A*) Mammalian cells are treated with 100 µM 4sU for 15 h to achieve maximum 4sU incorporation into RNA. (*B*) Exposure to 365 nm UV light crosslinks RNA to interacting proteins. (*C*) Cells are lysed and (*D*) the lysate is treated with RNase to make proteins more accessible for immunoprecipitation. (*E*) Protein of interest is immobilized on the magnetic beads via immunoprecipitation. The RNA–protein complexes are treated with RNase to obtain short RNA fragments to map the protein binding site with high resolution. RNA fragments are dephosphorylated for the subsequent DNA-adapter ligation. (*F*) 3′ Ligation of the preadenylated DNA adapter. For the visualization of the RNA–protein complexes, the IR800CW dye is azide-conjugated to the 3′end of the adapter. (*G*) The RNA–protein complexes are eluted from the beads, resolved on the SDS–PAGE, and visualized in the infrared channel. The RNA–protein complexes are eluted from the gel and the protein is digested with the proteinase K. RNA fragments are recovered with acidic phenol and size selected on the TBE-Urea gel. (*H*) RNA fragment is reverse transcribed from the primer complementary to the adapter sequence. The resulting ssDNA is purified on the TBE-Urea gel and circularized in a circular ligation reaction. (*I*) The library is amplified in PCR reaction introducing Illumina barcode and adapter sequences. The library schema is shown in detail in Supplemental Figure 1A.

Our IR-PAR-CLIP protocol mainly follows earlier established protocols with several modifications (Fig. 1). To allow efficient crosslinking, photoactivatable modified nucleoside 4sU is added to cell culture media 15 h prior to collection, to allow for incorporation into the cellular RNAs (Fig. 1A). Cells are exposed to 365 nm UV light to crosslink the 4sU-containing RNAs with interacting proteins (Fig. 1B), collected and lysed (Fig. 1C). Clarified cell lysate is treated with RNase for initial fragmentation of the RNAs. At this step RNase treatment facilitates the IP of the protein of interest and reduces the contamination from other RBPs interacting with the same mRNA (Fig. 1D). For the IP of the endogenous protein, here we used an anti-IGF2BP3 antibody coupled to protein G magnetic beads. Following the IP, a second RNase treatment is performed while the crosslinked protein–RNA complexes are still coupled to the beads. This treatment further shortens the RNA footprints to map the RBP-binding sites with high resolution. As both RNase I and RNase T1 used in this work leave a 2′,3′-cyclic phosphate, the crosslinked RNA footprints have to be dephosphorylated to allow 3′ adapter ligation (Fig. 1E). Next, the preadenylated IR-dye-conjugated DNA adapter is ligated to the RNA fragments (Fig. 1F; Supplemental Fig. 1A). The IR-dye allows visualization of ligated fragments at attomolar amounts (Zarnegar et al. 2016) and the random sequence at the 5′ end of the adapter helps to reduce the ligation bias (König et al. 2010; McGlincy and Ingolia 2017). The protein–RNA-adapter complexes are then eluted from the beads and resolved on SDS–PAGE which is visualized at a near-infrared light imager (here, LI-COR Odyssey CLx). The protein–RNA-adapter complexes are size selected on a gel (Supplemental Fig. 1B), extracted from the gel fragments, and treated with proteinase K to digest the crosslinked protein leaving the small peptide remnant. The peptide–RNA-adapter complexes are then size-selected on a denaturing RNA gel. The efficiency of RNase treatment can be already estimated during size selection (Fig. 1G; Supplemental Fig. 1C). Next, the purified peptide–RNA-adapter complexes are reverse transcribed with the primer containing flexible hexa-ethyleneglycol spacer. It allows efficient circular ligation and prevents the rolling-circle amplification during final library amplification (Ingolia 2010; McGlincy and Ingolia 2017). The cDNA is separated from the unreacted primer and no-insert products on a denaturing gel (Fig. 1H; Supplemental Fig. 1D) and ligated in a circular ligation reaction. The cDNA library circles are then amplified and indexed in a library construction PCR and separated from the excess of the primer on a gel (Fig. 1I; Supplemental Fig. 1E). The detailed step-by-step protocol is attached as Supplemental Material, the library schema is shown in Supplemental Figure 1A, and the most crucial optimization steps including the effect of RNase treatment on RBP targets identification are described below.

### Optimization of the immunoprecipitation of the endogenous IGF2BP3

**FIGURE 2.**
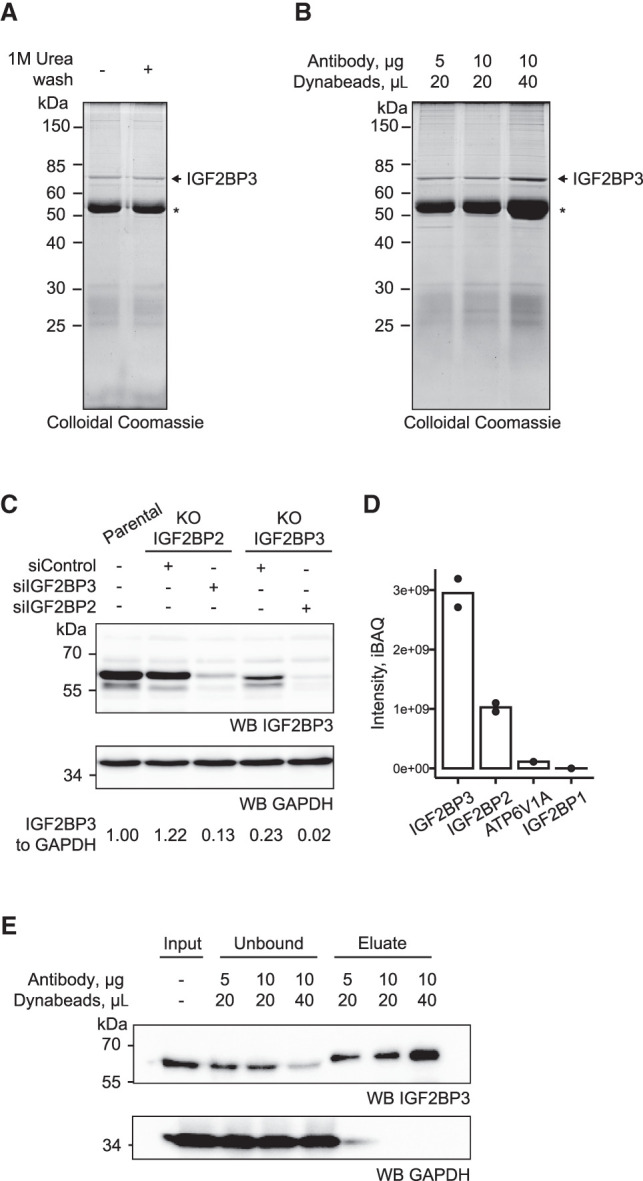
Optimization of immunoprecipitation of endogenous IGF2BP3. (*A*,*B*) Eluates from the immunoprecipitation reactions of IGF2BP3 from 25 mln HCT116 cells (∼2.5 mg of total protein) with Proteintech (14642-1-AP) anti-IGF2BP3 antibody coupled to protein G Dynabeads at indicated amounts resolved on SDS–PAGE and stained with a Colloidal Coomassie. * IgG heavy chain. (*C*) Western blot of the IGF2BP2 and IGF2BP3 CRISPR–Cas9 knockout HCT116 treated with the siRNA against IGF2BP3 and IGF2BP2, respectively, to deplete the remaining paralog. (*D*) Mass spectrometry intensities in the eluate of the immunoprecipitation of IGF2BP3 with Proteintech (14642-1-AP) anti-IGF2BP3 antibody. (*E*) Western blot of the IP described in (*B*). The input, unbound fraction, and eluates were loaded at a 1:1:1 ratio.

The CLIP methods rely on immunoprecipitation of the protein of interest for the isolation of specific RBP–RNA complexes from cells. For higher efficiency and obtaining cleaner IPs, tagging the protein of interest has been a common strategy. The addition of a tag to the protein of interest can affect protein levels and function, and the selection of the tag and its position in the protein sequence may require optimization. The availability of IP-compatible antibodies recognizing the protein of interest allows the characterization of the protein in various cell types at its endogenous levels and without manipulating its native structure. Earlier studies indicated that tagging IGF2BP3 might impact its association with polysomes and was suggested to impact its function (Bell et al. 2013). To be able to map the interaction of endogenous IGF2BP3 with its targets in mammalian cells, we characterized the IP-compatible antibody from Proteintech (14642-1-AP). As the PAR-CLIP approaches involve the purification of the crosslinked RBP–RNA complexes from gels, the purity of the IP samples is highly crucial. We performed the IPs in the presence of high salt for stringency. The colloidal Coomassie staining of the SDS–PAGE gel of the IP eluates revealed that Proteintech anti-IGF2BP3 antibody showed a single major band corresponding to the size of IGF2BP3 (Fig. 2A). It is advisable to visualize the gels with highly sensitive protein staining approaches in addition to western blotting to assess the purity of the eluates for PAR-CLIP experiments.

When isolating the endogenous protein, the specificity of the antibody has to be tested to ensure that it does not recognize other proteins with similar molecular weights. One of the most important challenges of the PAR-CLIP experiments for endogenous IGF2BP3 is the high sequence conservation between IGF2BP paralogs. Therefore, the cross-reactivity of the polyclonal antibody has to be considered. We used the HCT116 colorectal carcinoma cell line that has low levels of IGF2BP1, but high IGF2BP2 expression (Mongroo et al. 2011; Nusinow et al. 2020; Lu et al. 2021). To test whether the anti-IGF2BP3 antibody recognizes IGF2BP2, we analyzed HCT116 CRISPR–Cas9 knockouts of IGF2BP2 and IGF2BP3. By using siRNA depletion of IGF2BP2 in IGF2BP3 KO HCT116 cells, we found that the Proteintech anti-IGF2BP3 antibody partially recognizes IGF2BP2 resulting in ∼20% contamination which has to be considered when interpreting the data (Fig. 2C). We corroborated these results by performing mass spectrometry analyses following IP of IGF2BP3 using the Proteintech antibody (Fig. 2D; Supplemental Table 1). As IGF2BP paralogs form RNA-bridged complexes, we performed the RNase treatment prior to the IPs and applied extensive high salt washes similar to our PAR-CLIP experiments. The peptide intensity of the next most abundant contaminant ATP6V1A protein [catalytic subunit of the V1 complex of vacuolar(H+)-ATPase] was more than 100 times lower than that of IGF2BP3 and, as the protein has no known RNA-binding activity, it should not crosslink to RNA.

Isolating most of the protein from cell lysates is crucial as the unbound portion of the protein might be present in complexes that are not as accessible to IP, and an incomplete IP might affect the interpretation of the data. For the IP experiments, we coupled the antibody to magnetic beads (Dynabeads, Thermo Fisher). We tested the ratio of Dynabeads to antibody to identify conditions that capture the highest amount of IGF2BP3 from cell lysates (Fig. 2B,E). Using 4 µg antibody for 16 µL of Dynabeads per 1 mg of total protein within the lysate resulted in ∼75% depletion of the protein and was selected for further experiments (Fig. 2B,E). Based on these results, we decided to use Proteintech anti-IGF2BP3 antibody for our subsequent experiments.

### The infrared labeled 3′ adapter provides a robust and sensitive alternative to radioactive labeling

**FIGURE 3.**
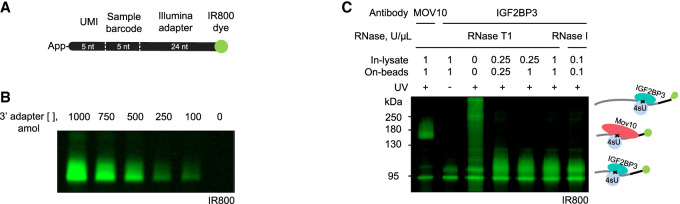
Infrared adapter ligation allows visualization of RNA–protein complexes with high sensitivity. (*A*) Schematic of the DNA adapter. The preadenylated DNA adapter contains the UMI, sample barcode, and Illumina adapter sequence. The IR800CW dye is azide-conjugated to the 3′end of the adapter. (*B*) The infrared adapter can be detected at the TBE-Urea gel starting from 100 attomoles (∼1 pg DNA). (*C*) SDS–PAGE of the RNA–protein complexes after infrared adapter ligation. Immunoprecipitation of endogenous MOV10 (114 kDa) or IGF2BP3 (64 kDa) proteins was done in 4sU-crosslinked lysates. The control without crosslinking shows a gel background. The crosslinked RNA-adapter fragments add ∼30–50 kDa to the apparent molecular weight of the protein depending on the RNase treatment conditions.

Traditionally, radioactive isotopes have been used to visualize crosslinked protein–RNA complexes and RNA fragments at various steps of CLIP protocols. The use of radioactive isotopes presents challenges in the widespread application of the method. They represent a health hazard and require a high safety level laboratory space. Moreover, radioactive isotopes decay causing a variation of the signal across experiments. To overcome these challenges, we used an infrared dye labeling strategy for PAR-CLIP experiments. We used IR800CW (LI-COR) as it provides high sensitivity with low fluorescence background. A similar strategy has been successfully implemented in other CLIP methods (Zarnegar et al. 2016; Kaczynski et al. 2019). In our protocol, we used the 5′ preadenylated 3′ DNA adapter labeled at the 3′ end with an azide-conjugated infrared dye. 5′ adenylation of the adapter and blocking of the 3′ end of the sequence with the azide-conjugated dye ensures the single direction of ligation (Fig. 3A; Supplemental Fig. 1A). The described 3′ adapter contains the following features: (i) Illumina adapter sequence, (ii) 5-nt unique molecular identifier (UMI) that is used to remove the PCR duplicates during analysis, and (iii) 5-nt index sequence. Adapter-ligated RNAs containing different index sequences can be pooled together before the reverse transcription reaction (König et al. 2010).

The infrared detection of the IR800CW dye displayed high sensitivity, as 100 attomoles of the adapter visualized on the gel displayed sufficient signal intensity for detection (Fig. 3B). The SDS–PAGE of crosslinked protein–RNA-adapter complexes showed little background and displayed distinct bands for the two proteins we tested based on their different molecular weights (MOV10 [114 kDa] and IGF2BP3 [64 kDa]). The crosslinked RNA-adapter fragments add ∼30–50 kDa to the apparent molecular weight of the protein depending on the size of the RNA conjugate at different RNase treatment conditions. The appearance of RNase-sensitive high molecular weight products confirmed that the observed complexes result from the adapter-crosslinked RNA footprints (Fig. 3C). Additionally, those gels allowed us to estimate the efficiency of RNase cleavage and the yield of the isolated complexes. Altogether, we established a modified IR-PAR-CLIP protocol that allowed us to visualize crosslinked RNA–protein conjugates with high sensitivity.

### RNase treatment impacts the identification of IGF2BP3-binding sites and mRNA targets

In the PAR-CLIP method, RBP–RNA complexes are subjected to RNA fragmentation by RNase treatment at two distinct steps: (i) RNase treatment of the lysate to decrease copurification of additional RBPs in RNA-bridged RNP complexes and (ii) RNase treatment on beads to allow for size selection. To fragment the crosslinked RNAs in CLIP experiments, RNase I and RNase T1 are most commonly used. The sequence preferences of RNases and differences in digestion efficiency can introduce a bias into the PAR-CLIP data and affect conclusions. While RNase I has a low sequence preference, RNase T1 preferentially digests after guanosines and can therefore introduce a stronger bias (Kishore et al. 2011; Gerashchenko and Gladyshev 2017). Interestingly, although PAR-CLIP produces short reads that are more prone to be affected by the RNase sequence preferences, most PAR-CLIP protocols use RNase T1 (Hafner et al. 2010; Friedersdorf and Keene 2014; Danan et al. 2016), while RNase I is more commonly used in iCLIP and eCLIP protocols (König et al. 2010; Conway et al. 2016; Van Nostrand et al. 2016; Buchbender et al. 2020)

**FIGURE 4.**
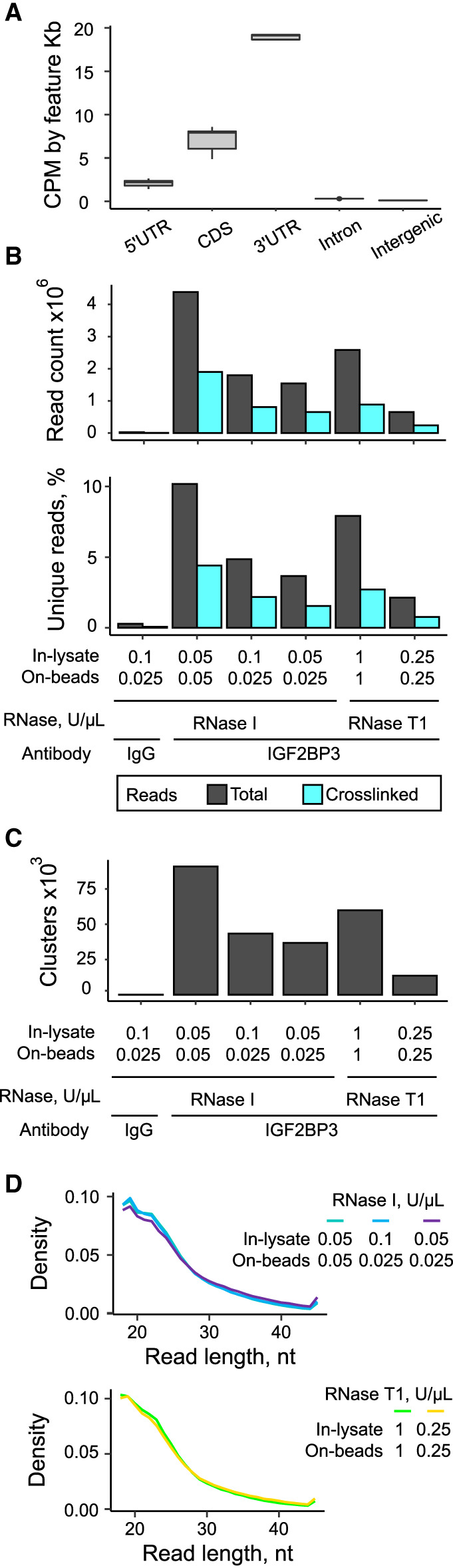
Effect of RNase treatment conditions on the recovery of crosslinked RNA fragments. (*A*) Read alignment of the libraries made in this study to genome features. (*B*) Counts and percentages of the unique deduplicated reads in the final libraries depending on RNase treatment conditions. (*C*) The number of clusters identified with PARalyzer depending on RNase treatment conditions. (*D*) Distribution of lengths of deduplicated reads within the 18–45 nt range.

To systematically address the effect of RNase cleavage on the results of IGF3BP3 PAR-CLIP, we tested RNase T1 and RNase I at several concentrations. In all of the libraries, the reads preferentially mapped to the 3′UTR regions in agreement with known IGF2BP3-binding preferences in cancer cells (Fig. 4A; Hafner et al. 2010; Palanichamy et al. 2016; Huang et al. 2018) and the IGF2BP3 libraries contained on average 2000 times more crosslinked read clusters compared to the IgG control sample (Fig. 4B,C). In PAR-CLIP, two RNase treatment steps are usually performed: first treatment in the lysate intended to partially digest the RNA, facilitate the IP and reduce the co-IP of other RBPs bound to the same mRNA and the second treatment after the IP to shorten the RNA footprints for better identification of the binding motifs. For RNase I, we tested the in-lysate concentrations of 0.05 and 0.1 U/µL with a constant on-beads concentration of 0.025 U/µL and the on-beads concentrations of 0.05 and 0.025 U/µL with a constant in-lysate concentration of 0.05 U/µL. In this concentration range, we observed that the increase of the RNase concentration both in-lysate and on-beads improved the yield of the unique reads and the number of identified clusters (Fig. 4B,C) while the lower RNase concentrations only slightly increased the lengths of the aligned deduplicated reads (Fig. 4D). Similarly, the combination of RNase T1 concentrations of 1 U/µL in-lysate and on-beads resulted in higher numbers of the aligned reads and identified clusters than 0.25 U/µL (Fig. 4B–D). The efficiency of RNase treatment depends on multiple parameters including cell type, the nature of interactions between the protein of interest and its targets, and the concentration of this protein in the cells. Therefore, optimization of the RNase treatment for a particular experiment may be required.

**FIGURE 5.**
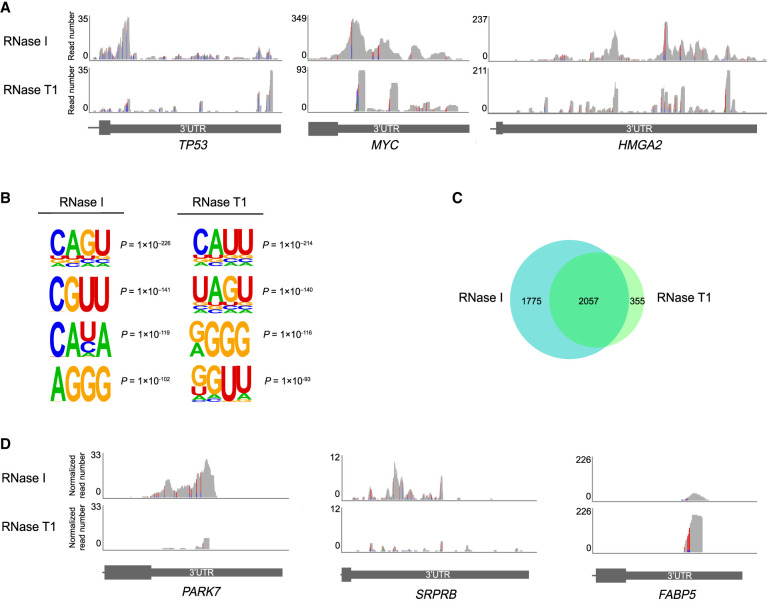
RNase selection affects IR-PAR-CLIP coverage, motif and target prediction results. (*A*) Examples of IGF2BP3 PAR-CLIP read coverage of 3′UTRs of *TP53*, *MYC*, and *HMGA2* transcripts. (*B*) 4-nt IGF2BP3-binding motifs identified by HOMER in IR-PAR-CLIP of HCT116. (*C*) Overlap of IGF2BP3 targets identified in this study with RNase I and RNase T1. Concentrations of RNases used for this figure in-lysate and on-beads: RNase I 0.05 U/µL, RNase T1 1 U/µL. (*D*) Examples of IGF2BP3 PAR-CLIP library-size normalized read coverage of 3′UTRs of *PARK7*, *SRPRB*, and *FABP5* transcripts. Transitions color code: (A) green, (C) blue, (G) brown, (T) red.

To reveal whether treatment with various RNases impacts the results, we compared the PAR-CLIP coverage for IGF2BP3 obtained with RNase I and RNase T1. Strikingly, the coverage profiles on 3′UTRs differed for multiple targets. The representative examples of *TP53*, *MYC*, and *HMGA2* show that RNase I digestion results in broader coverage than RNase T1 and can possibly identify different binding sites (Fig. 5A). Indeed, the analysis of overrepresented 4-nt motifs identified CAGU as the most significant motif for each RNase I concentration tested (Fig. 5B; Supplemental Fig. 2A). This sequence was not identified in RNase T1-treated samples where the strongest motif was CAUU in agreement with previously published IGF2BP3 PAR-CLIP data obtained with RNase T1 digestion (Fig. 5B; Supplemental Fig. 2A; Hafner et al. 2010).

Moreover, RNase I treatment allowed us to identify ∼1.5 times more IGF2BP3-bound targets compared to RNase T1 including most of the targets identified with RNase T1 (Fig. 5C; Supplemental Fig. 2B; Supplemental Table 2). Interestingly, highly intersecting sets of targets were identified in all RNase I concentration conditions despite having up to a two-times difference in the number of aligned crosslinked deduplicated reads (Fig. 4B) or the number of identified clusters (Fig. 4C). The number of targets identified with RNase I increased with increasing RNase I concentration. In contrast, a similar number of targets was identified with the two tested RNase T1 treatment conditions (1 or 0.25 U/µL) although the number of aligned crosslinked deduplicated reads in 1 U/µL condition was ∼4 times higher than in 0.25 U/µL (Fig. 4B), as was the number of the identified clusters (Fig. 4C). To exclude the possibility that the higher number of targets obtained in samples with the RNase I treatment resulted from the different number of useful reads, we compared the results of the samples treated with RNase T1 at 1/1 U/µL with the ones treated with RNase I 0.1/0.025 U/µL. At those concentrations, RNase T1 produced a slightly higher number of aligned crosslinked deduplicated reads and identified clusters compared to RNase I, but the number of identified targets was still 1.4 times higher in RNase I sample. Most of the targets that we identified only in RNase I conditions were not found in RNase T1 due to lower coverage at the cluster (less than 10 counts per million [CPM]) (e.g., *PARK7*, *SRPRB*; Fig. 5D) and several examples had less than 50% of T to C transitions at the covered positions (e.g., *FABP5*; Fig. 5D). Although the number of targets identified using two RNase T1 concentrations was similar, more than 30% of the targets were identified only in one of the conditions. This can be explained by the RNase T1 cleavage bias resulting in pronounced differences in the recovered footprints for samples treated with different enzyme concentrations. The differences between the RNase treatment conditions were also evident in the Pearson correlation matrix of the IGF2BP3 IR-PAR-CLIP coverage (Supplemental Fig. 2C), with RNase I conditions showing higher correlation coefficients compared to RNase T1. Altogether, our results revealed that RNase I treated samples yielded a higher number of IGF2BP3 RNA targets with higher confidence.

### IR-PAR-CLIP identifies novel IGF2BP3 RNA targets in colon cancer cell lines

IGF2BP3 expression is elevated in colon cancers and was shown to be associated with increased tumor angiogenesis, growth, and poor prognosis (Lochhead et al. 2012; Shantha Kumara et al. 2015; Xu et al. 2019; Yang et al. 2020). Several mechanisms were suggested for the function of IGF2BP3 in promoting tumorigenesis in colon cancers including regulation of mRNA localization, translation, and stabilization of oncogenic transcripts (Rivera Vargas et al. 2014; Deforzh et al. 2016; Ennajdaoui et al. 2016; Li et al. 2020). Therefore, thorough identification of IGF2BP3 targets in colon cancers is important for understanding its function.

**FIGURE 6.**
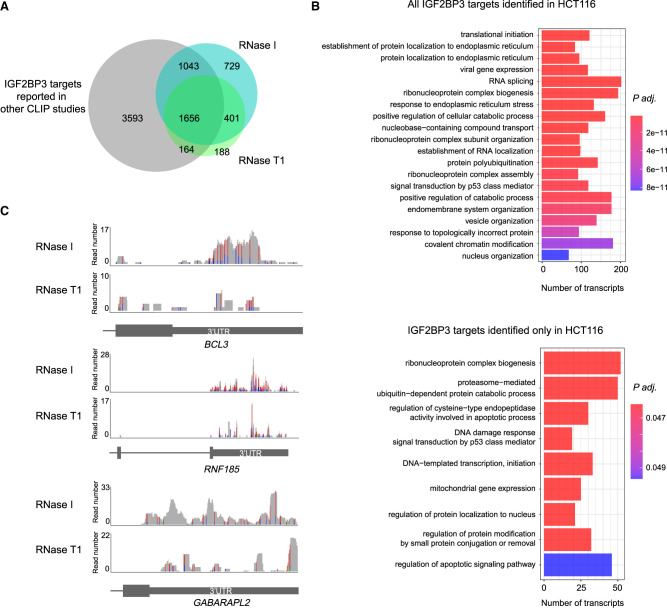
Novel IGF2BP3 RNA targets identified by IR-PAR-CLIP in HCT116 colorectal carcinoma cell line. (*A*) Overlap of IGF2BP3 targets identified in this study with RNase I and RNase T1 with the aggregated list of IGF2BP3 targets identified in the HEK 293 (Hafner et al. 2010), human pluripotent stem cells (Conway et al. 2016), B-ALL (Palanichamy et al. 2016), hepatocellular carcinoma (HepG2) (ENCODE [Dunham et al. 2012]), and pancreatic ductal adenocarcinoma (PL45 and Panc1) (Ennajdaoui et al. 2016). Concentrations of RNases used for this figure in-lysate and on-beads: RNase I 0.05 U/µL, RNase T1 1 U/µL. (*B*) Gene Ontology (GO) term analysis of biological process (BP) categories enriched in HCT116 targets identified in this work with both RNase I and RNase T1 (HCT116 all) or when the previously identified targets (A) are excluded (HCT116 only). For visualization, the GO enrichment results were simplified by clustering using the *simplify* function from the clusterProfiler R package. (*C*) Examples of IGF2BP3 PAR-CLIP read coverage of 3′UTRs of *BCL3*, *GABARAPL2*, and *RNF185* transcripts. Transitions color code: (A) green, (C) blue, (G) brown, (T) red.

In this work, we identified the mRNA targets of IGF2BP3 in the colorectal carcinoma cell line HCT116 using our optimized IR-PAR-CLIP method. We compared our results obtained with both RNase I and RNase T1 (4181 targets) with IGF2BP3 targets identified by the PAR-CLIP method in HEK293 (Hafner et al. 2010), by other CLIP approaches in human pluripotent stem cells (Conway et al. 2016), B-acute lymphoblastic leukemia (B-ALL) (Palanichamy et al. 2016), hepatocellular carcinoma (HepG2) (ENCODE [Dunham et al. 2012]), and pancreatic ductal adenocarcinoma (PL45 and Panc1) (Ennajdaoui et al. 2016) (6456 targets) (Supplemental Table 2). This comparison revealed 2863 common targets identified in the analyzed cell types and 1318 novel targets only identified in HCT116 cells (Fig. 6A). The IGF2BP3 target pool might differ depending on cell line-specific factors, the most prominent factor being differences in transcriptome composition and the expression level of transcripts. Therefore, our results highlight the importance of the identification of IGF2BP3 targets in various cancers in order to reveal their role in tumorigenesis.

The GO term analysis of all the IGF2BP3 targets in HCT116 cells showed that the IGF2BP3-bound transcripts belong to multiple functional categories (biological process) involved in mRNA processing and metabolism, RNA localization and targeting to the endoplasmic reticulum, response to proteotoxic stress and chromatin modifications (Fig. 6B; Supplemental Table 3). These categories were highly similar to previously published data indicating that IGF2BP3 interacts with a core set of RNAs independent of the tissue or cell type. Interestingly, the 1318 targets characteristic only for HCT116 included transcripts playing a role in DNA-damage response and apoptotic pathway regulation (e.g., *CDK4*, *BAX*, *CASP2*, *BCL3*, *BCL10*), proteasome-mediated degradation (e.g., *USP7*, *CUL3*, *TRIM2*, *PSMA6*, *PSMA5*, *PSMD8*), and autophagy (e.g., *LAMTOR1*, *GABARAPL2*) and included numerous components of transcription initiation mediated by DNA polymerases I and II (e.g., *POLR1A*, *POLR1B*, *POLR1C*, *POLR2D*, *MED4*, *MED14*, *MED20*, *MED24*, *TAF4*, *TAF9*) (Fig. 6B,C) suggesting that IGF2BP3 might be involved in the regulation of these processes in colon cancer.

## DISCUSSION

Emerging data reveal the importance of posttranscriptional mechanisms in cellular adaptation and development (Becker et al. 2018; Rendleman et al. 2018). Multiple RBPs bind and regulate the fate of different mRNA targets depending on the cellular state and developmental stage. This has been linked to the differences in mRNA abundance, RNA modifications, and posttranslational modification of the RBPs as well as the interactions of RBPs with other proteins (Hüttelmaier et al. 2005; Hafner et al. 2013; Huang et al. 2018). Therefore, identifying mRNA targets bound to RBPs at different cellular, metabolic, or developmental states is of crucial importance. Identifying the RNAs that are directly bound to the RBP of interest is challenging. Isolating ribonucleoprotein complexes from cells requires stringent conditions, which might lead to the loss of transient interactions between RBP and RNAs (Riley and Steitz 2013). To identify RNAs interacting with the RBP of interest and map the binding sites with high confidence, CLIP methods are widely used (Lee and Ule 2018; Hafner et al. 2021). Those methods rely on crosslinking of RNAs to the RBP of interest in their native environment in cells before their isolation from cells by IP. Originally, UV light at 254 nm has been used for crosslinking approaches. However, due to the low efficiency of crosslinking at this wavelength, PAR-CLIP approaches relying on the introduction of photoactivatable nucleosides have been developed (Hafner et al. 2010; Danan et al. 2016).

The IGF2BP family of RBPs plays an important role in posttranscriptional regulation by controlling mRNA localization, stability, and translation (Nielsen et al. 1999; Ladd et al. 2001; Fallini et al. 2011; Mizutani et al. 2016). IGF2BP3 is one of the three paralogs of the IGF2BP family. Due to its high expression in aggressive tumors and its role in regulating the stability of mRNAs that promote cell growth and metastasis, IGF2BP3 has been widely studied. According to previously published data (Dunham et al. 2012; Conway et al. 2016; Palanichamy et al. 2016) and our current study (Fig. 6A), IGF2BP3 binds to multiple mRNAs and the RNA target pool can vary dramatically depending on the cell type similar to many other RBPs (Van Nostrand et al. 2020). In this paper, we developed and applied a modified PAR-CLIP protocol IR-PAR-CLIP for the identification of transcripts interacting with endogenous IGF2BP3 (Fig. 1).

In IR-PAR-CLIP, we optimized or modified four important steps of the protocol. (i) We replaced radioactive isotopes with IR fluorescence tags to visualize the RNA fragments. (ii) We developed a streamlined approach to test antibody specificity for IGF2BP paralogs and optimized the IP protocol for human IGF2BP3. (iii) We implemented circular ligation to the PAR-CLIP protocol to increase efficiency and reduce sequence bias during library preparation. (iv) We tested the biases introduced by the RNases used to fragment the RNAs during PAR-CLIP and optimized RNase cleavage. Altogether, using our optimized IR-PAR-CLIP method, we revealed 1318 novel RNA targets of IGF2BP3s in colon cancer cells with high confidence.

The CLIP approaches rely on the IP of the RBP–RNA complexes from cells to identify their direct target RNAs. IP of the RBP of interest using specific antibodies against the protein is an attractive approach that does not require the attachment of affinity tags to the RBP of interest. Even though the CRISPR–Cas9 gene editing methods have become more reliable, efficient, and accessible, ensuring homozygous insertion of the tag to both alleles of the gene of interest requires clonal selection and careful further characterization of various clones, which still poses a challenge. Moreover, the addition of the tag may alter protein expression levels, localization, and function. Thus, performing IPs with antibodies against the protein of interest is preferable if such antibodies are available. In this case, the specificity of the antibody is of crucial importance as contamination with another RBP may impact the experimental outcome. We found that visualizing the IP eluates via unbiased sensitive protein staining methods such as colloidal Coomassie allows careful assessment of the specificity of the antibodies (Fig. 2A). Another possible challenge when performing IPs for endogenous proteins is the affinity of the antibody of interest for paralogs of the RBP. Many RBPs have closely related paralogs in mammalian cells including IGF2BP3. The sequence identity among the IGF2BP paralogs is ∼73%. To test the specificity of the anti-IGF2BP3 antibody, we used IGF2BP3 and IGF2BP2 knockout cell lines together with siRNA depletion of the second paralog (Fig. 2C). Moreover, we performed mass spectrometry analyses with the eluates of the IPs using the anti-IGF2BP3 antibody. We found that the anti-IGF2BP3 antibody partially recognizes the IGF2BP2 paralog and in the MS analyses IGF2BP2 might cover a maximum of 20% of the total reads obtained with IGF2BP3 antibody (Fig. 2D). In the future, the use of monoclonal antibodies raised against the diverging sequences might help to increase the selectivity of antibodies for IGF2BP3 paralogs.

In the PAR-CLIP method, the crosslinked RBP–RNA complexes are subjected to size selection at distinct stages to further purify the complexes and increase the stringency in the protocol. Traditionally, the RBP–RNA complexes and the isolated fragments have been visualized by radioactive isotopes using autoradiography. Even though highly sensitive, this method is tedious due to the need for permits, the possibility of contamination, and the long exposure times of radioactive labels. Moreover, due to the decay of the radioactive isotopes the signal varies from experiment to experiment. Here, we optimized the use of IR fluorescence tags to label the RNAs at the 3′ end by the use of IR800-dye labeled adapters. The IR-labeled 3′ adapter could be visualized at a 100 attomole range (Fig. 3B). According to our estimations, ∼2 femtomole of IGF2BP3- or MOV10-RNA-3′adapter complexes could be isolated from 25 million HCT116 cells and visualized with more than sufficient intensity (Fig. 3C). Our results suggest that IR-PAR-CLIP offers a sensitive, reliable, and convenient alternative to radioactive isotopes to visualize RNAs at different steps of the PAR-CLIP protocol.

The low input amounts present a challenge in the CLIP methods. To increase the efficiency of the library preparation, recent protocols have used reverse transcriptases with high processivity and optimized the size selection and the reaction clean-up steps to find effective ways to get rid of the excess of the unreacted adapter, RT primer, and no-insert products (Zarnegar et al. 2016; Van Nostrand et al. 2017; Lee and Ule 2018; Buchbender et al. 2020; Anastasakis et al. 2021). In order to increase the efficiency in IR-PAR-CLIP, here we used highly processive reverse transcriptase Superscript IV and implemented the on-bead ligation of a preadenylated 3′ DNA adapter. We used the adapter sequence that contains the UMIs and index sequences allowing deduplication and multiplexing of samples (König et al. 2010). To additionally increase the efficiency and to reduce the ligation bias, we implemented a circular ligation strategy using the reverse transcription primer with an optimized sequence containing the carbon linker which facilitates circular ligation and prevents rolling circle amplification (Ingolia 2010; König et al. 2010; McGlincy and Ingolia 2017). Recently, omitting the RNA size selection step and performing the size selection during the first step of the two-step PCR approach was shown to increase the efficiency of PAR-CLIP library preparation (Anastasakis et al. 2021). We did not implement this change to the current protocol as we used the denaturing RNA gels to control for the efficiency of the RNase cleavage and separate the ligated RNA fragments from the longer non-crosslinked RNAs and no-insert products. Additionally, the separation of longer, potentially non-crosslinked RNAs on a denaturing gel allowed us to omit the transfer to the nitrocellulose membrane, as this step was stated to decrease the efficiency of the recovery of the crosslinked fragments (Anastasakis et al. 2021). In future protocols, if the RNA size selection is omitted, the use of ssDNA exonuclease in combination with 5′ deadenylase may be beneficial to reduce the contamination with the unreacted adapter as described in McGlincy and Ingolia (2017). To further increase the efficiency of the protocol, the bead-based size selection methods can be optimized for purifying the reverse transcription product, as implemented in eCLIP (Van Nostrand et al. 2016) and iCLIP2 protocols (Buchbender et al. 2020). With the described IR-PAR-CLIP approach, we were able to amplify the IGF2BP3 PAR-CLIP library obtained from 125 million cells at 16 cycles. The final library contained ∼10% of aligned deduplicated reads and resulted in the identification of 80,000 IGF2BP3-binding clusters and ∼4000 mRNA targets. We observed that different RNA fragmentation conditions lead to variations in the number of identified clusters within one order of magnitude and are, therefore, an important step in the protocol that requires optimization.

Optimization of the RNase treatment in the PAR-CLIP is crucial as too short reads will not allow unique genomic mapping while too long reads complicate the precise mapping of the binding sites. Importantly, different nucleases display preferences in their cleavage sequence, and this can introduce biases when one is assigning the binding sites (Kishore et al. 2011). Even though micrococcal nuclease (MNase), RNase A, and RNase I have been implemented in some PAR-CLIP protocols, currently RNase T1 is the most frequently used nuclease for PAR-CLIP experiments in the literature (Kishore et al. 2013; Danan et al. 2016; Garzia et al. 2017; Anastasakis et al. 2021). In CLIP approaches, RNase T1 was shown to introduce a sequence bias, because it preferentially cleaves after guanosines (Haberman et al. 2017). RNase I, which does not have any described nucleotide preferences, was often used in recent CLIP papers (Conway et al. 2016; Haberman et al. 2017; Lee and Ule 2018). Here, we tested whether RNase I or RNase T1 treatment impacts the number of identified targets as well as the sequences identified by IR-PAR-CLIP of IGF2BP3 (Figs. 4, 5). We optimized limited RNase digestion for the two RNases and subjected the IGF2BP3 IR-PAR-CLIP libraries to next-generation sequencing. We found that in samples treated with RNase I, there were around 3800 RNA targets identified in our analyses. In contrast, only approximately 2500 RNAs were identified when libraries were prepared using RNase T1. Importantly, around 2000 of those RNAs were common between RNase I and T1 treated samples indicating that RNase I treatment does not fail to recover targets. Most of the targets that did not pass the selection criteria in RNase T1-treated samples displayed a too low number of reads per cluster compared to the samples subjected to RNase I treatment. Moreover, we also observed that T to C transition sites were not covered for some targets to pass the selection criteria during the analyses. In summary, RNase I outperformed RNase T1 in the identification of IGF2BP3 targets in colon cancer cells.

Intriguingly, the IGF2BP3 interaction motifs identified for RNase I and RNase T1 treated samples showed distinct differences in the top four most reliably predicted motifs. Upon RNase T1 treatment, the motif with the highest score, CAUU, was identical to that identified earlier for IGF2BP3 in HEK293 cells following RNase T1 treatment (Hafner et al. 2010). Instead, in HCT116 cells treated with RNase I, the top scoring motif was CAGU (Fig. 5B; Supplemental Fig. 2A). The motifs obtained with RNase T1 might be depleted of some G-containing sequences if the RNA is digested inside of the recognition motif. Therefore, for PAR-CLIP protocols RNase treatment conditions have to be selected carefully with the preference to be given to the RNases that have low sequence specificity, like RNase I.

Our IR-PAR-CLIP method allowed us to identify 1318 novel mRNAs bound to IGF2BP3s in a colorectal carcinoma cell line (HCT116). Many of these genes are involved in the regulation of cell cycle, apoptosis, and proteostasis and have cancer-associated functions, suggesting that by regulating their stability or translation IGF2BP3 might contribute to cancer progression (Fig. 6A). For many RBPs, the identity of the targets highly depends on the cell type and condition, therefore the identification of RBP targets in various cellular contexts using the most sensitive methods is crucial for understanding their function and mechanism of action. We anticipate that our framework can be used to identify the targets of other RBPs in a variety of cell types or tissues.

## MATERIALS AND METHODS

### IR-PAR-CLIP

The crosslinking, proteinase treatment, and size selection parts of the protocol are based on the published PAR-CLIP protocol (Hafner et al. 2010; Danan et al. 2016), the ligation of the IR 3′ adapter is adapted from irCLIP methods (Zarnegar et al. 2016), and the library preparation strategy from iCLIP (König et al. 2010) and ribosome profiling protocols (Ingolia et al. 2009; Ingolia 2010; McGlincy and Ingolia 2017). The detailed step-by-step protocol is provided in the Supplemental Material.

### Preadenylation of the 3′ adapter and conjugation of the infrared dye

The 3′ adapter was ordered as RNase-Free HPLC purified oligonucleotide phosphorylated at the 5′ end and containing azide (NHS ester) at the 3′ end with the following sequence: /5Phos/NNNNNATCGTAGATCGGAAGAGCACACGTCTGAA/3AzideN/ (McGlincy and Ingolia 2017). A total of 250 pmol of the oligo was preadenylated in a 50 µL reaction using the 5′ DNA Adenylation Kit (NEB) according to the manufacturer's instructions. The reaction was incubated at 65°C for 2 h. Then the oligonucleotide was purified using Oligo Clean & Concentrator columns (Zymo), and IRdye-800CW-DBCO (LiCor) was conjugated via “click” chemistry at 37°C for 2 h as described in Zarnegar et al. (2016) and Kaczynski et al. (2019).

### Cell culture

HCT116 conditionally expressing Tet-OsTIR1 were obtained from the Masato Kanemaki lab (Natsume et al. 2016). The cells were tested for mycoplasma contamination and mycoplasma contamination was not detected. The HCT116 cells were cultured in McCoy's 5A (modified) medium (Sigma) with 10% fetal bovine serum (Gibco), 2 mM glutamine (Sigma), 1% Pen/Step (Sigma). 4sU (Sigma) was added to the cell culture media 15 h prior to collection at 100 µM. For IR-PAR-CLIP, five 15 cm (diameter) dishes of cells per condition were plated with 3.5 million HCT116 cells per dish so that they reach 60% confluency at the time of collection, resulting in approximately 125 million cells per condition. Cells were put on ice and washed with 10 mL of ice-cold PBS (Sigma). PBS was completely aspirated and 1 mL of PBS was added to prevent cells from drying. Crosslinking was performed with the UV light at 365 nm with 0.15 J/cm^2^. Upon crosslinking, cells were collected via scraping, then were pelleted, frozen in liquid nitrogen, and stored at −80°C.

### Cell lysis, in-lysate RNase treatment, and immunoprecipitation

Cell pellets from 125 million cells per condition were lysed in 1250 µL of ice-cold lysis buffer (25 mM HEPES pH 7.3, 150 mM NaCl, 0.5% NP-40, 0.5 mM EDTA, 10% glycerol, 0.1% SDS, 0.2% sodium deoxycholate, 1× protease inhibitors cocktail, 0.1 mM DTT), by incubation on ice for 15 min with intermittent vortexing and passing three times through the 27G needle. The lysate was clarified by centrifugation for 20 min at 20,000*g* at +4°C. A total of 1100 µL of the supernatant was taken for the in-lysate RNase digestion on a rotator for 15 min at room temperature. The RNase I (Ambion) was used at 0.1 or 0.05 U/µL and RNase T1 (Thermo Scientific) at 1 or 0.25 U/µL. For IP, 50 µg of Proteintech anti-IGF2BP3 antibody (14642-1-AP, lot 00090203) or Proteintech IgG control (30000-0-AP) was coupled to the 200 µL of protein G Dynabeads (Invitrogen) in 1 mL of lysis buffer for 20 min, rotating at room temperature, washed three times with 1 mL of the lysis buffer, resuspended in the original bead volume (200 µL) and added to 1 mL of the RNase treated lysate. The IP was incubated on a rotator at +4°C for 4 h, washed twice, and resuspended in 1 mL of the lysis buffer. To test the sensitivity of protein-crosslinked-RNA-3′ adapter fragments to RNase, one dish of HCT116 per condition was used as described above and all reaction volumes were scaled down proportionally. MOV10 IP was performed using Proteintech anti-MOV10 antibody (10370-1-AP, lot00001954).

### On-beads RNase treatment, dephosphorylation, 3′ adapter ligation, and SDS–PAGE of protein–RNA complexes

For the on-beads, RNase treatment RNase I (Ambion) was added at 0.05 or 0.025 U/µL and RNase T1 (Thermo Scientific) at 1 or 0.25 U/µL. The combinations of in-lysate and on-beads RNase concentrations are indicated in the Results section. For RNase treatment, the samples were incubated on the rotator for 15 min at room temperature and cooled on ice for 5 min. Then the beads were washed three times with 1 mL of ice-cold high salt wash buffer (25 mM HEPES pH 7.3, 400 mM NaCl, 0.5% NP-40, 0.5 mM EDTA, 10% glycerol, 1× protease inhibitors cocktail, 0.1mM DTT), with 3 min incubations on ice after each wash step. For dephosphorylation of the crosslinked RNA fragments, the beads were washed once with 1 mL of PNK wash buffer (20 mM Tris-HCl pH 6.5, 10 mM MgCl_2_, 0.2% Tween20, 0.1 mM DTT), resuspended in 200 µL of dephosphorylation mix (1× PNK buffer pH 6.5 [70 mM Tris-HCl pH 6.5, 10 mM MgCl_2_, 1 mM DTT], 100 U T4 polynucleotide kinase [NEB], 20 U SUPERase·In [Invitrogen]) and incubated at 37°C for 30 min with shaking at 1100 rpm. Afterward, the beads were kept on ice and washed once with 1 mL of PNK wash buffer, incubated in 1 mL of high salt wash buffer for 5 min on ice, and washed once more with 1 mL of PNK wash buffer. For 3′ adapter ligation, the beads were resuspended in 200 µL of the ligation reaction mix (20% PEG8000, 1× T4 RNA ligase reaction buffer [NEB], 1000 U T4 RNA ligase 2 truncated KQ [NEB], 50 pmol 5′ preadenylated IR-dye-conjugated DNA adapter, and 20 U SUPERase·In) and incubated at 25°C for 3 h with shaking at 1100 rpm in the dark. Next, the beads were transferred on ice and washed once with 1 mL of PNK wash buffer, incubated in 1 mL of high salt wash buffer for 5 min on ice, resuspended in 1 mL of PNK wash buffer, and transferred to the new low-bind RNase-free 1.5 mL tube. The PNK wash buffer was removed, and the crosslinked protein–RNA complexes were eluted in 40 µL of 1× SDS sample buffer without DTT at +70°C for 10 min. DTT at 20 mM concentration was added to the collected eluates, and the samples were heated at +70°C for 10 min and stored at −80°C. Next, the crosslinked protein–RNA complexes were resolved on a 10-well 4%–12% NuPAGE Novex Bis-Tris gel (Invitrogen) in 1× MOPS-SDS running buffer (Invitrogen) at 200 V for 65 min. The gel was visualized at 800 nm using LI-COR Odyssey CLx near-infrared imager, and RBP–RNA complexes were size selected using the imager's grid. The bands corresponding to IGF2BP3–RNA complexes were excised from the gel and transferred to a low-bind RNase-free tube.

### Proteinase K digestion, 3′ adapter-ligated RNA extraction, and size selection

For the samples with the RNase treatment conditions in-lysate and on-beads: RNase I 0.05 U/µL, RNase T1 1 U/µL proteinase K digestion was performed as described in Acosta-Alvear et al. (2018). The gel piece was transferred to a nuclease-free water preequilibrated Pur-A-Lyzer Midi Dialysis Tube (MWCO 3.5 kDa, Sigma), with 400 µL 1× MOPS-SDS running buffer (Invitrogen), and electroelution was performed at 100 V for 2 h. The polarity of the electric current was reversed for 120 sec, the sample was transferred to the new 2 mL low-bind RNase-free tube with an equal volume of 2× Proteinase K buffer (100 mM HEPES pH 7.3, 100 mM NaCl, 20 mM EDTA, 2% SDS) and proteinase K (Invitrogen) at a final concentration of 1.2 mg/mL, and incubated for 30 min at +55°C. For the samples with RNase treatment conditions: RNase I 0.05 U/µL in-lysate and 0.025 U/µL on-beads, RNase I 0.01 U/µL in-lysate and 0.025 U/µL on-beads, and RNase T1 0.25 U/µL in-lysate and on-beads gel extraction and protein digestion were performed as described in Anastasakis et al. (2021). Gel pieces were transferred to a 1.5 mL low-bind RNase-free tube, frozen at −80°C, thawed at +55°C and crashed with a single-use RNase-free pellet pestle. Gel slurry was boiled in 2× proteinase K buffer with 50 mM DTT at +95°C for 2 min and was subsequently incubated three times with fresh addition of proteinase K at 2.4 mg/mL, 1.5 mg/mL, and 2.4 mg/mL in 2× proteinase K buffer at +50°C for 30 min with shaking at 1100 rpm. Extracted 3′ adapter-ligated RNA fragments were separated from the gel by centrifugation at 10,000*g* for 10 min through a Spin-X centrifuge filter (Costar). For all samples, RNA was extracted with 1 mL of acidic phenol–chloroform–isoamyl alcohol (25:24:1, pH 4.0), isopropanol precipitated, resuspended 1× TBE-Urea dye (Invitrogen), denatured by heating at +75°C for 2 min, and resolved on a 10-well 15% TBE-Urea gel (Invitrogen) in 1× TBE running buffer (Invitrogen) at 180 V for 70 min. The gel was visualized at 800 nm using LI-COR Odyssey CLx near-infrared imager and the 3′ adapter-ligated RNA fragments were size selected using the imager’s grid.

### Reverse transcription, circular ligation, library construction PCR, and sequencing

Gel pieces were transferred to a 1.5 mL low-bind RNase-free tube, frozen at −80°C, thawed at +55°C, and crashed with a single-use RNase-free pellet pestle. 3′ adapter-ligated RNA fragments were extracted from the gel during overnight incubation rotating in 300 µL of RNA elution buffer at +4°C (10 mM Tris-HCl pH 7.0, 0.3 M NaOAc pH 5.5, 2 mM EDTA, 20 U SUPERase·In). Extracted complexes were separated from the gel fragments by centrifugation at 10,000*g* for 10 min through a Spin-X centrifuge filter, ethanol precipitated and resuspended in 11.5 µL of nuclease-free water. For reverse transcription, 1 µL of 1 µM reverse transcription primer (/5Phos/RNAGATCGGAAGAGCGTCGTGTAGGGAAAGAG/iSp18/GTGACTGGAGTTCAGACGTGTGCTC) was added to the sample. The sample was incubated at +70°C for 10 min and transferred on ice. Then the components of the reverse transcription mix were added to the sample making a final volume of 20 µL (1× Superscript IV buffer, dNTPs 0.2 mM each, 5 mM DTT, 10 U SUPERase·In, 200 U Superscript IV [Invitrogen]); the reaction was performed at +55°C for 20 min and inactivated at +80°C for 5 min. A total of 2.2 µL of 1 M NaOH was added, and RNA was hydrolyzed in 30 min incubation at 98°C. The reaction was neutralized by the addition of 2.2 µL of 1 M HCl. The reverse transcription reaction was purified on Oligo Clean & Concentrator columns (Zymo) according to the manufacturer's instructions and eluted in 6 µL of nuclease-free water. cDNA was mixed with 6 µL of 2× TBE-Urea dye, denatured by heating at +75°C for 2 min, and resolved on a 10-well 10% TBE-Urea gel (Invitrogen) in 1× TBE running buffer at 180 V for 70 min. The gel was stained with SYBR Gold (Invitrogen) and visualized, and the reverse transcription product was separated from the unreacted primer and no-insert products. Gel extraction was performed similarly to the 3′ adapter-ligated RNA fragments using DNA elution buffer (10 mM Tris-HCl pH 8.0, 0.3 M NaOAc pH 5.5). After ethanol precipitation, the pellet was resuspended in 10 µL of circular ligation reaction mix (1× CircLigase II buffer, 2.5 mM MnCl_2_, 50 U CircLigase II [Lucigen]). Circular ligation was performed at +60°C for 2 h, and the enzyme was inactivated through heating at +80°C for 10 min. To determine the number of cycles required for the PCR amplification for the library construction, a series of small-scale PCR reactions was performed with NEB Phusion polymerase and index primers (NEB, E6609S). The reaction mix of 65 µL (1× High Fidelity buffer, dNTPs 0.2 mM each, 500 nM NEB Universal primer, 500 nM NEB Index primer, 2.8 µL circularization reaction, 1.3 U Phusion polymerase) was aliquoted to six PCR tubes (10 µL) and run according to the following program: 98°C, 30 sec; 20 cycles of 98°C, 10 sec; 65°C, 10 sec; 72°C, 10 sec. The tubes were removed at the end of extension at cycles 10, 12, 14, 16, 18, and 20 and the products were resolved on 10-well 6% TBE gel (Invitrogen) in 1× TBE running buffer at 180 V for 40 min and stained with SYBR Gold. For the library construction PCR, the cycle number was selected where the bright product band appeared. Typically, the number of cycles was 16–18 for IGF2BP3 IP and 22–23 for IgG control. The reaction composition and conditions were the same as for the PCR designed to determine the cycles; additionally, the final extension was performed for 5 min at 72°C. Libraries were ethanol precipitated, resolved on a 10-well 6% TBE gel similarly to the cycle selection PCR, and separated from the no-insert library and unreacted primers. Gel extraction was performed similarly to the extraction after the reverse transcription reaction, but the gel pieces were not heated higher than +30°C. After ethanol precipitation, pellets were resuspended in 10 µL of nuclease-free water. Library size distribution and quantity were determined using the Agilent Bioanalyzer 2100 with the High Sensitivity DNA Kit (Agilent) and via qPCR with the NEBNext Library Quant Kit for Illumina (NEB). Libraries were sequenced on a NovaSeq 6000 S1 at SR100 mode (Illumina) at the Vienna BioCenter NGS facility producing 55 million reads per sample on average.

### Computational analysis

For the demultiplexed data sets, UMIs were extracted and adapter sequences were trimmed using UMI-tools v1.1.1 (Smith et al. 2017). The reads were size and quality trimmed using Trimmomatic v0.30 (Bolger et al. 2014) to have a length between 18 and 45 nt. The reads then were mapped to the GENCODE human genome assembly GRCh38.p13 with bowtie v0.12.7 (Langmead et al. 2009), allowing up to three mismatches and deduplicated using UMI-tools v1.1.1. IGF2BP3-binding clusters were called with PARalyzer v1.5 (Corcoran et al. 2011) (ini file is available in Supplemental Material). Genes that have at least one cluster containing more than 10 CPM and more than 50% of T to C conversions per read were selected as IGF2BP3 targets. The enrichment of 4-nt motifs in IGF2BP3-binding clusters was analyzed with HOMER v4.11 (Heinz et al. 2010), findMotifsGenome.pl command using exome as a background. The Pearson correlation between the IGF2BP3 IR-PAR-CLIP conditions was calculated for the 50-nt coverage windows using the multiBamSummary and plotCorrelation tools from deepTools (Ramírez et al. 2016). Sequencing data processing was conducted using the HPC of the Center for Integrative Bioinformatics Vienna (CIBIV), Austria. The published lists of IGF2BP3 targets were obtained from Conway et al. (2016), Palanichamy et al. (2016), and Ennajdaoui et al. (2016). The ENCODE eCLIP IGF2BP3 target list was downloaded from the POSTAR3 (Zhao et al. 2022) (source data: ENCODE project [ENCSR993OLA], Dunham et al. 2012). For data from Conway et al. (2016), the gene was selected as IGF2BP3 target if CDS or 3′UTR peak coverage was four times higher than in SMIinput in IGF2BP3 eCLIP, and not more than two times higher in the IgG control. The IGF2BP3 PAR-CLIP data (GSM545209, SRR048962) (Hafner et al. 2010) were analyzed in parallel with the data from the current study with the same parameters used for bowtie alignments, peak calling, and target identification. GO term analysis was performed with clusterProfiler v3.18.1 (Yu et al. 2012).

### Optimization of IGF2BP3 immunoprecipitation

To test the IGF2BP3 immunoprecipitation conditions, one 15 cm (diameter) dish of 60% confluent HCT116 (∼25 million cells) per condition was washed in ice-cold PBS, scraped, pelleted, and resuspended in 250 µL of ice-cold lysis buffer. Cells were lysed by incubation with the lysis buffer on ice for 15 min with intermittent vortexing and passing the cell suspension three times through the 27G needle. The lysate was clarified by centrifugation for 20 min at 20,000 and treated with 0.2 U/µL RNase I (Ambion) rotating at room temperature for 15 min. For IP, from one 15 cm (diameter) dish 5 µg of Proteintech (14642-1-AP, lot 00090203) antibody was coupled to Dynabeads, as described before in 1 µg:4 µL antibody:beads ratio. The lysates were rotated at +4°C for 4 h for the IP. The flowthrough was removed using a magnetic rack, and the IP-ed complexes were washed five times in one volume (250 µL) of ice-cold high salt wash buffer with 3-min incubations on ice. For the urea wash, urea was added to the buffer during the first wash to a final concentration of 1 M. Protein was eluted in 25 µL of sample buffer as described in the section “On-beads RNase treatment, dephosphorylation, 3′ adapter ligation, and SDS–PAGE of protein–RNA complexes” above. The eluate was resolved on SDS–PAGE in tris-glycine SDS buffer and analyzed with colloidal Coomassie staining (Dyballa and Metzger 2009) or western blotting with anti-IGF2BP3 (Proteintech, 14642-1-AP) and anti-GAPDH (Proteintech, 10494-1-AP) antibodies.

### Establishment of IGF2BP2 and IGF2BP3 knockout cell lines and siRNA depletion

For knockout cell line generation, gRNA sequences (IGF2BP2: 5′GAGCTGCCGGAGGTCGTCGG 3′; IGF2BP3: 5′ACGCGTAGCCAGTCTTCACC 3′) were cloned into the pSpCas9 (BB)-2A-GFP (PX458) (plasmid #48138; Addgene) (Ran et al. 2013). Cells were transiently transfected using jetOPTIMUS reagent (Tamar, 101000051), and GFP-positive single-cell clones were FACS sorted at BD FACSAria IIIu at Max Perutz Labs BioOptics FACS Facility. For siRNA knockdown, cells were transfected with ON-TARGETplus Human IGF2BP2 (Dharmacon, L-017705-00-0005) or IGF2BP3 (Dharmacon, L-003976-00-0005) SMARTpool siRNAs using DharmaFECT 2 (Dharmacon, T-2002-01). ON-TARGETplus nontargeting siRNA #1 (Dharmacon, D-001810-01-05) was used as a control.

### Mass spectrometry of IGF2BP3 immunoprecipitation

For mass spectrometry of IGF2BP3 immunoprecipitation, three 15 cm (diameter) dishes of 60% confluent HCT116 per condition were washed in ice-cold PBS, scraped, pelleted, and resuspended in 750 µL of ice-cold lysis buffer. The IP was performed as described in the section “Optimization of IGF2BP3 immunoprecipitation” above using 30 µg of Proteintech anti-IGF2BP3 antibody (14642-1-AP, lot 00090203) per sample. RNA was digested using 1 U/µL RNase T1 (Thermo Scientific). Protein was eluted in 50 µL of sample buffer, resolved on a 10-well 4%–12% NuPAGE Novex Bis-Tris gel (Invitrogen) in 1× MOPS-SDS running buffer (Invitrogen), and stained with colloidal Coomassie (Dyballa and Metzger 2009). The band corresponding to IGF2BP3 was cut from the gel, submitted for tandem mass spectrometry, and analyzed with MaxQuant 1.6.17.0 at the Max Perutz Labs Mass Spectrometry Facility.

## DATA DEPOSITION

All raw and processed sequencing data generated in this study have been deposited in the Gene Expression Omnibus database, https://www.ncbi.nlm.nih.gov/geo/ (accession no. GSE229653).

## SUPPLEMENTAL MATERIAL

Supplemental material is available for this article.

## Figures and Tables

**Aleksandra Anisimova:**
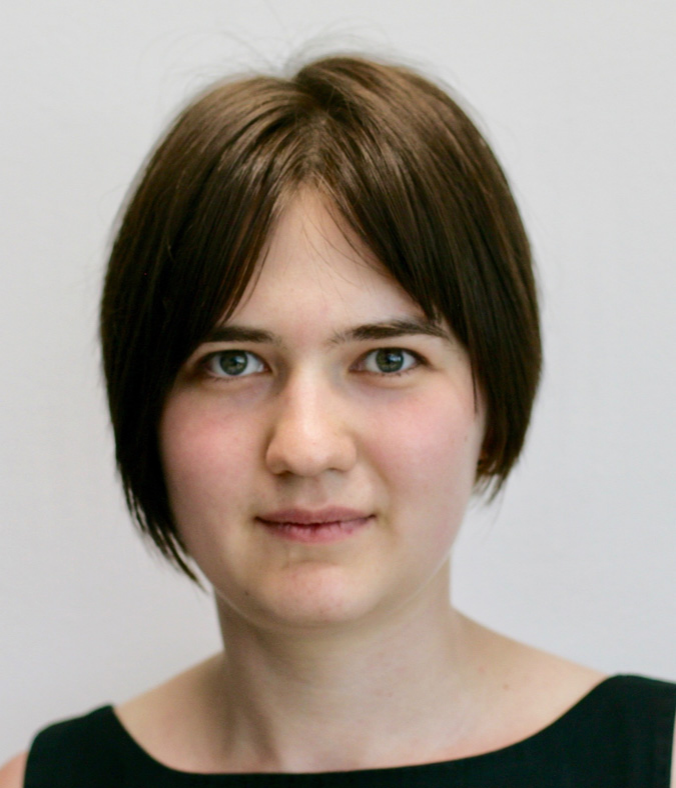


## MEET THE FIRST AUTHOR

***Meet the First Author(s)* is an editorial feature within *RNA*, in which the first author(s) of research-based papers in each issue have the opportunity to introduce themselves and their work to readers of *RNA* and the RNA research community. Aleksandra Anisimova is the first author of this paper, “Optimized infrared photoactivatable ribonucleoside-enhanced crosslinking and immunoprecipitation (IR-PAR-CLIP) protocol identifies novel IGF2BP3-interacting RNAs in colon cancer cells.” Anisimova is a PhD student in the Vienna BioCenter (VBC) who is doing research in the group of Elif Karagöz at Max Perutz Laboratories, as a joint venture of the University of Vienna and the Medical University of Vienna in Austria. The main focus of Anisimova's research is understanding the role of posttranscriptional regulation in the cellular response to proteotoxic stress, particularly focusing on mRNA stability and translation regulation.**

**What are the major results described in your paper and how do they impact this branch of the field?**

In the current paper, we describe the infrared photoactivatable ribonucleoside-enhanced crosslinking and immunoprecipitation (IR-PAR-CLIP) protocol, which enables genome-wide identification of the RNA regions interacting with a specific RNA-binding protein (RBP). We demonstrate this using the endogenous IGF2BP3 in the HCT116 colon carcinoma cell line as an example. Crosslinking and immunoprecipitation experiments are complex and include several steps where the selection of the right strategy or the treatment conditions is crucial. In this study, we paid particular attention to optimizing conditions for the immunoprecipitation of the protein of interest and conditions for the RNase treatment. Our findings show that identified RNA targets, coverage profiles, and the RNA-binding motifs of IGF2BP3 were strongly influenced by the type of RNase used. We also further optimized and modified specific steps in the PAR-CLIP protocol. Compared to previous versions of the PAR-CLIP protocol, the IR-PAR-CLIP uses an incredibly sensitive infrared dye that enables the visualization of the RBP-bound RNAs at the attomolar range. Moreover, we have used a circular ligation strategy previously reported to minimize the ligation bias in the small RNA sequencing libraries. Using the IR-PAR-CLIP, we identified the novel IGF2BP3-bound transcripts in the colon carcinoma cell line involved in several biological processes including DNA-damage response, transcription initiation, proteasome-mediated degradation, and autophagy.

**What led you to study RNA or this aspect of RNA science?**

I suppose that every reader of the *RNA* journal will agree that RNA is an extremely fascinating molecule with a list of functions that are hard to count. It is elegant, flexible, has enormous regulatory potential, and may even be responsible for the origin of life itself. I clearly recall the moment that sparked my interest in the world of RNA biology. It was an exam during my bachelor's studies where the students were asked to name as many RNA classes as possible. The answer was accepted if the list contained more than 15 correct classes, and clearly the actual number is much higher. This functional diversity seemed very fascinating to me. Of course, for a student to start his or her path as a scientist, simple curiosity is not enough. Their development as a researcher depends on the chosen laboratory and project, and I believe that at this point one also needs a bit of luck. In my case, I was fortunate to meet an incredible mentor, Dr. Sergey Dmitriev, who supported my interest in RNA biology, taught me careful laboratory work, and guided me through my thesis project, which addressed several aspects of the regulation of translation and the biology of the ribosome. My enthusiasm to study RNA at the next career stages was further strengthened by the incredible RNA community and exciting conferences that covered the broad spectrum of the functions of RNA.

**During the course of these experiments, were there any surprising results or particular difficulties that altered your thinking and subsequent focus?**

The results of the current study, along with previously published work, demonstrate that IGF2BP proteins bind to thousands of transcripts. However, a much smaller number of these transcripts are actually regulated by these proteins, and this regulation depends strongly on the biological context. Initially, it was hard for me to accept that even strong binding of IGF2BP to the transcript does not necessarily result in regulation. Understanding of the molecular mechanisms behind this conundrum has become one of the directions of my PhD research.

**If you were able to give one piece of advice to your younger self, what would that be?**

During their training, students are often reminded of the importance of asking the right question for a scientist. I heard this statement repeatedly and tried to keep it in mind when formulating my research goals. However, it was just one of many considerations. Looking back, I think if I could invite my younger self for a cup of coffee, I would try my best to convince myself that an accurate research question is not just important, but actually imperative for a successful experiment and project. Planning a network of experiments in your mind and carefully considering the potential outcomes and strategies are the skills that scientists can and should develop. It's helpful to remember that curiosity, while a driving force in science, can also be a distraction if not managed well and should not be followed blindly. One should ask, “Is this experiment actually necessary to answer my research question?” Most of the time, if the answer is no, it is better to set it aside and return to it later when it may provide exciting details on the discovered mechanism.

**Are there specific individuals or groups who have influenced your philosophy or approach to science?**

As a bachelor's, master's, and PhD student, I had the opportunity to learn from and work with amazing teachers and scientists who mentored, supported and challenged me. Each of them influenced my understanding of science, for which I am deeply grateful. One of the most important things for me was to see that researchers have a variety of different approaches to science. In this regard, I would also like to mention the book entitled *Candid science II: conversations with famous biomedical scientists*, by Professor Istvan Hargittai, that I read during my high school years. It contained engaging interviews with prominent biochemists and molecular biologists of the 20th century and showed the diversity of opinions and work styles among them. Over the past eight years, I've been fortunate to be supervised by four exceptional scientists: my master's thesis supervisors Dr. Sergey Dmitriev, Professor Vadim Gladyshev, and Dr. Ivan Kulakovskiy and my PhD project supervisor, Dr. Elif Karagöz. They encouraged my determination to tackle particularly challenging biological questions, emphasized the necessity of extreme care and attention to detail, and demonstrated the power of integrating genome-wide methods with focused biochemical approaches.
